# Does opportunity co-creation help the poor entrepreneurs? Evidence from China

**DOI:** 10.3389/fpsyg.2023.1093120

**Published:** 2023-02-10

**Authors:** Xiaoting Chen, Yanling Zheng, Haiquan Chen, Yu Tian

**Affiliations:** ^1^School of Journalism and Communication, South China University of Technology, Guangzhou, China; ^2^School of Management, Guilin University of Aerospace Technology, Guilin, China; ^3^School of Management, Jinan University, Guangzhou, China; ^4^School of Business, Sun Yat-sen University, Guangzhou, China

**Keywords:** opportunity co-creation, opportunity belief, enterpreneurial action, enterpreneurial performance, enterpreneureship, poverty alleviation

## Abstract

Extreme poverty can be alleviated through entrepreneurship, but starting a business can be elusive among impoverished people, partly due to a lack of access to entrepreneurial opportunities. In the current literature, the source of entrepreneurial opportunity for the poor remains unclear. To address this knowledge gap, we used the opportunity co-creation perspective to examine the impact of opportunity co-creation on the entrepreneurial performance of the poor and its various influence pathways. We developed a chain multiple mediation model and surveyed 330 poor entrepreneurs from the Wuling Mountain Region, which used to be one of the 14 contiguous poverty-stricken areas in China until the end of 2020 when the country announced the eradication of extreme poverty. Data analysis was done using structural equation modeling (SEM). The results suggest that opportunity co-creation has a direct positive effect on the entrepreneurial performance of the poor and an indirect positive effect through the chain mediating effect of opportunity beliefs and entrepreneurial behavior. The findings confirm that opportunity co-creation is a critical factor for entrepreneurs in poor areas to overcome the lack of entrepreneurial opportunities and can also contribute to a better understanding of opportunity beliefs and entrepreneurial behavior. Furthermore, these results have important implications for poor entrepreneurs and provide opportunity co-creation solutions for poverty reduction through entrepreneurship.

## 1. Introduction

Entrepreneurship has been viewed as a crucial tool to alleviate extreme poverty (Sutter et al., 2019). Researchers in management and other disciplines have continually explored reducing poverty through entrepreneurship. However, not everyone would be able to succeed in entrepreneurship (Sutter et al., 2019). The current literature is unable to explain the variability of entrepreneurial performance, particularly among the poor (Wu et al., 2020). This leads us to ask: what factors determine the entrepreneurship performance of poor people? Given that opportunity is the key to entrepreneurship research (Shane and Venkataraman, 2000), we looked for answers by examining entrepreneurial opportunities.

Since the emergence of entrepreneurship studies, many scholars have widely shared the idea of entrepreneurial opportunity core (Shane and Venkataraman, 2000). However, the source of entrepreneurial opportunities remains controversial in the current literature (Davidsson, 2015). There are two contradictory epistemologies: the discovery view (entrepreneurial opportunities are discovered by entrepreneurs) and the construction view (entrepreneurial opportunities are constructed by entrepreneurs; Alvarez et al., 2013). In recent years, some have blended these two perspectives, suggesting that entrepreneurial opportunities can be both discovered and constructed. According to this perspective, entrepreneurs can explore and construct entrepreneurial opportunities in multiple ways (Si et al., 2015). However, either epistemology has mostly focused independently on factors such as entrepreneurs, entrepreneurial firms, and the external environment. This has caused the independent functions and roles of factors to be magnified (Dimov, 2007a; McKelvey et al., 2015), while the interaction between multiple factors in creating entrepreneurial opportunities has been largely ignored (Busenitz et al., 2003; Johnson and Schaltegger, 2020).

Uncertain circumstances and limited knowledge, among other things, often prevent poor entrepreneurs from creating markets for their inventive ideas. These markets often do not exist or are imperfect (Alvarez and Barney, 2014). Entrepreneurship among the poor emphasizes the traits and subjectivity of the entrepreneurial subject (Nakara et al., 2021), as well as the impact of the entrepreneurial environment (Alvarez et al., 2015). As a result, the source of its entrepreneurial opportunities is distinct. Entrepreneurship among the poor is influenced not only by their human capital (Alvarez and Barney, 2014), financial capital (Sutter et al., 2019), and spatial surroundings (Lazos-Ruíz et al., 2014) but also by their institutional environment (Wu et al., 2022b). The entrepreneurial opportunities of the poor are not just the objective formation of the environment or the subjective creation of the entrepreneurial subject but also exist in the relationship between the entrepreneurial subject and the entrepreneurial environment. This means that entrepreneurial opportunities are influenced and continuously shaped by the interactions between the direct environment and the social environment. Is there a new epistemology that may explain the source of entrepreneurial opportunities for the poor?

To further explore the concept of entrepreneurial opportunities, this study reviewed prior research on entrepreneurial opportunities from the three theoretical perspectives, i.e., opportunity discovery, opportunity creation, and opportunity integration, and investigated the limitations in the existing literature. Taking into account the specificity of the poor’s entrepreneurial opportunity source, we integrated opportunity discovery view and opportunity creation view, and proposed an opportunity co-creation view. Although the concept of entrepreneurial discovery or construction can explain the characteristics of the poor and the enstrepreneurial behavior of external partners, it does not fully reveal how personal traits, external partners, and the entrepreneurial environment interact with the formation of entrepreneurial opportunities (Alvarez et al., 2015).

The entrepreneurial process involves beliefs, actions, and results (BAR; Foss and Klein, 2020). Opportunity belief refers to the individual belief formed by entrepreneurs after overcoming doubts about opportunity information (Shepherd et al., 2007). Opportunity belief is an emerging research topic in the field of entrepreneurship. Current research has concentrated more on the mechanism behind the formation of the entrepreneurs’ opportunity beliefs (Pidduck et al., 2021). In poor areas with high uncertainty, the window of opportunity is short-lived, threatened by rapid technological progress and fierce competition (Qin et al., 2017; Han et al., 2022). Whether entrepreneurial ideas can be successfully transformed into entrepreneurial opportunities, the role of opportunity beliefs cannot be ignored. Establishing a belief in opportunity can help poor entrepreneurs overcome their doubts and encourage entrepreneurial actions. The formation of opportunity beliefs marks entrepreneurial boundaries and is a crucial stage in developing entrepreneurial cognition and changing entrepreneurial action (McMullen and Shepherd, 2006).

Additionally, as a key milestone in entrepreneurial research (McMullen and Shepherd, 2006), although numerous studies have been conducted on entrepreneurial actions, there is still room for further discussion. The majority of the literature has confirmed the influence of entrepreneurial action on entrepreneurial outcomes (Bird and Schjoedt, 2017). However, as part of the entrepreneurial process, few scholars have analyzed the formation mechanism of entrepreneurial actions in depth based on the perspective of opportunity co-creation.

In this study, we aimed to widen the research viewpoint on entrepreneurship and poverty reduction by examining how the co-creation of entrepreneurial opportunities may drive the poor to launch a business and improve entrepreneurial performance. In other words, entrepreneurial performance might be the outcome of collaboration between the impoverished and external partners. Our findings emphasize the critical contributions of the poor and the stakeholders in entrepreneurship through joint utilization and creation of entrepreneurial resources. We developed and tested a model that demonstrates how the co-creation of opportunities between the poor and government agencies may increase opportunity beliefs, promote the development of entrepreneurial behavior, and improve the entrepreneurial performance of the poor.

The key concern addressed in this study is how opportunity co-creation affects the entrepreneurial performance of the poor. We created a chain multiple mediation model, with opportunity co-creation as the independent variable, opportunity beliefs and entrepreneurial actions as double mediating variables, and entrepreneurial performance as the outcome variable. An empirical analysis was conducted using data on poor entrepreneurs in the Wuling Mountain region in China (N = 330) to answer the following two questions: What impact does opportunity co-creation have on entrepreneurial performance? How does opportunity co-creation affect entrepreneurial performance?

## 2. Literature review and hypothesis

### 2.1. Entrepreneurial opportunity

Entrepreneurial opportunities are situations in which new goods, services, raw materials, markets, and organizing methods can be introduced through the formation of new means, ends, or means-ends relationships (Shane and Venkataraman, 2000; Eckhardt and Shane, 2003). Numerous studies have demonstrated that entrepreneurial opportunity is the heart of entrepreneurship (McMullen and Shepherd, 2006; Plummer et al., 2007; Suddaby et al., 2015) and continues to be the primary problem in entrepreneurship (Shane and Venkataraman, 2000). While the core theory of entrepreneurial opportunities has been widely explored in recent years, there remains a fierce academic discussion over the origin and generation of entrepreneurial opportunities. There are three forms of analyses (Alvarez et al., 2013; Suddaby et al., 2015): discovery view (Kirzner, 1997; Shane, 2012), construction view (Sarasvathy et al., 2005; Alvarez and Barney, 2007), and discovery and construction view (Dyer et al., 2008; Si et al., 2015).

Scholars with a view of discovery argue that entrepreneurial opportunities do not depend on the will of entrepreneurs but exist objectively in external contexts (Shane and Venkataraman, 2000; Dutta and Crossan, 2005) that are discovered by alert entrepreneurs (Kirzner, 1997). This point of view emphasizes the decisive factors of the external environment (De Carolis and Saparito, 2006; Vermeire and Bruton, 2016). Under the opportunity discovery framework, a goal-oriented logic of action is displayed by entrepreneurs who set goals in advance around potential market opportunities and translate abstract goals into concrete entrepreneurial actions by selecting appropriate means (Farrokhnia et al., 2022).

Scholars holding the constructive perspective argue that entrepreneurial opportunities are endogenous and do not exist objectively but are instead constructed by entrepreneurs based on their unique and creative concepts and change perceptions of the external environment (Alvarez and Barney, 2007; Suddaby et al., 2015). Emphasizing human subjective perception and creativity (Chiasson and Saunders, 2005), this viewpoint believes that entrepreneurial opportunities are imagined and constructed by entrepreneurs through repeated reflection and understanding of the external environment and the interactions with other subjects (Steyaert and Hjorth, 2003; Grégoire et al., 2010a; Vermeire and Bruton, 2016). Under the opportunity construction framework, a means-oriented logic of action is displayed by entrepreneurs who change or create environmental conditions conducive to entrepreneurial interests based on available resources or means (Sarasvathy, 2001; Farrokhnia et al., 2022) to convince target customers that they need their innovation (Suddaby et al., 2015).

With the evolution of the entrepreneurial paradigm, some scholars have conducted research based on the integrated perspective of “discovery” and “construction”(Si et al., 2015). Scholars with an integrated perspective believe the discovery and construction viewpoints are complementary. These two views are only empirically meaningful when they are linked to specific entrepreneurial actions, which depend on the nature of the entrepreneur’s assumptions about the business environment (Alvarez and Barney, 2007). In other words, entrepreneurs can create entrepreneurial opportunities and discover them. Discovery and creation are not completely at odds with each other; they are just two different serendipities, where one dominates at different objective environments (Sarasvathy, 2001).

Based on the literature review on the generation of entrepreneurial opportunities, most studies on entrepreneurial opportunity sources focus on independent factors, such as entrepreneurs, start-ups, and the external environment. These studies amplify the functions of these independent factors (Dimov, 2007a; McKelvey et al., 2015) and largely overlook the interactions between the various participating elements in generating entrepreneurial opportunities (Auerswald and Dani, 2017). Entrepreneurial opportunities result from the interactions between entrepreneurs, markets, environments and other elements to create new “means-ends” relationships (Busenitz et al., 2003; Johnson and Schaltegger, 2020). These are discovered or created not only by individual entrepreneurs but also by diverse entrepreneurial subjects and elements in the entrepreneurial environment, either directly or indirectly (Eckhardt and Shane, 2003; Dimov, 2007a; Davidsson, 2015; Ramoglou and Gartner, 2022).

### 2.2. Opportunity co-creation

The co-creation concept originated from the theory suggesting that the firm and the customer jointly create value (Prahalad and Ramaswamy, 2004). Based on this, some scholars have extended the relationship to all stakeholders (Sun and Im, 2015), proposing that entrepreneurial opportunities need to be created with stakeholders (Alvarez et al., 2015) so that they can contribute to poverty alleviation (Sun and Im, 2015). Consistent with prior research, we define opportunity co-creation as the process in which entrepreneurs generate social and economic values through repeated interactions with multiple stakeholders in the entrepreneurial environment and jointly identify and solve social problems (Sun and Im, 2015). The stakeholders may include customers, governments, investors, and employees (Alvarez and Barney, 2014; Sun and Im, 2015). This definition highlights the objective existence of entrepreneurial opportunities, the subjective behavior of entrepreneurs and stakeholders, and the influence of the entrepreneurial environment on the formation of entrepreneurial opportunities (Alvarez et al., 2015; Davidsson, 2015).

The view of opportunity co-creation has been validated. Sun and Im (2015) explored the opportunity co-creation path by conducting pairwise analyses of the complicated relationships between stakeholders. Luk et al. (2005) investigated the influence of corporate stakeholders on the performance of start-ups and the relationship between entrepreneurs and stakeholders. They found that the relationship between stakeholders has a synergistic effect and can effectively improve enterprise performance. Based on the “view of creation,” Chiasson and Saunders (2005) proposed that the evolution of entrepreneurial opportunity generation is influenced by the interactions between entrepreneurs, stakeholders, and circumstances. This means that the opportunity co-creation perspective comprises the selection, participation, dialogue, and construction between the enterprise and various stakeholders, emphasizing that every stakeholder creates new value for the enterprise and can obtain benefits through opportunity co-creation.

While the success of poor entrepreneurs is inextricably linked to the support of stakeholders, different types of stakeholders can have varying influences on their decision-making. The government’s role, is particularly important to the growth of entrepreneurship among the poor. Compared with regular entrepreneurs, poor entrepreneurs face increased psychological, social, organizational, and institutional constraints (Kumar et al., 2022a). Since these constraints are interrelated, they would have to be analyzed collectively and comprehensively (Chikweche and Fletcher, 2017). Given their limited capabilities, poor entrepreneurs often have difficulties solving these constraints alone and require corporate stakeholder assistance (Calton et al., 2013). At the same time, the role of the government has increasingly become critical due to the general market failure caused by externalities, market information asymmetry, insufficient resources and other challenges of starting a business. The government can protect the interests of poor entrepreneurs by formulating appropriate policies and institutions and can improve the material, human, and social capital of the poor by providing public services (Kumar et al., 2022b). Additionally, in China, because of the advantages of national institutions and resources, the poor’s entrepreneurial behavior is closely related to national policies (Wu et al., 2022b), which has made this group heavily reliant on government support in starting a business. In this instance, the identification and creation of entrepreneurial opportunities are joint efforts of the poor and the government.

To lift almost 100 million rural residents out of poverty, the Chinese government has implemented targeted poverty alleviation since 2013 (Deng et al., 2022). To ensure that assistance falls correctly into poor villages and households, the Central Government of China implemented a top-to-bottom accountability approach, putting in place a strict responsibility structure. This required the various levels of secretaries (i.e., provincial, municipal, county, township, and village) to collaborate in poverty reduction efforts (Liu et al., 2018). The first secretaries and resident village cadres play an important role in implementing poverty alleviation policies, strengthening grassroots organizations, and serving the population’s needs; they have the heavy responsibility of implementing the anti-poverty measures to the “last kilometer.” Together with villagers with an entrepreneurial spirit, resident village cadres learn about entrepreneurship through repeated investigations and research, revising and discussing entrepreneurial ideas, searching for suitable industrial poverty alleviation projects, and going door to door to encourage other villagers to join and establish cooperation. They also combine multiple resources to provide entrepreneurs with microfinance, transfer payments, education, and training support (Zhou et al., 2018) and jointly establish rural cooperatives. Through institutional support, the government improves the entrepreneurial environment, resulting in more sources and opportunities in poor communities. Poor entrepreneurs can frequently interact with the institutional environment through dialogue with government officials, research, and other practical activities, jointly discovering and creating entrepreneurial opportunities (Deng et al., 2022). With the improvements in the entrepreneurial situation, poor entrepreneurs can fully transform from “consumers” to “producers,” increase their income levels, and improve their quality of living, thus prompting sustainable growth in the local economy.

### 2.3. Opportunity co-creation and entrepreneurial performance

Entrepreneurial performance refers to the efficiency of the entrepreneurs’ input and output of resources under certain conditions (Laskovaia et al., 2017), which is the embodiment of the competitive advantages and outcomes of the business operations. The co-creation behavior of the poor and the government can help identify and develop entrepreneurial opportunities and improve the poor’s entrepreneurial performance. As the starting point of entrepreneurship, opportunity cognition and identification are crucial elements and sources for enhancing entrepreneurial performance (Alvarez and Barney, 2014; Guo et al., 2017). Opportunity is critical for companies to gain competitive advantages and perform well (Gielnik et al., 2012). If companies do not actively seek out and discover opportunities, they will struggle to survive and achieve success (Sambasivan et al., 2009; Anwar et al., 2022).

Opportunity recognition has been emphasized as a key strategy for the success of new ventures (Anwar et al., 2022). Identifying opportunities has a profound impact on the entrepreneurial performance of poor entrepreneurs. However, most of the poor live in remote areas (e,g., rural communities). Because of their geographical isolation, they struggle to keep up with market changes and lack basic entrepreneurial resources (Ring et al., 2010). In addition, their lack of formal education and comprehensive knowledge (Anderson and Obeng, 2017), limits the discovery, recognition, and creation of opportunities. In this situation, entrepreneurial opportunities need to be generated by poor entrepreneurs through dynamic interactions with various stakeholders (De Silva and Wright, 2019; De Silva et al., 2020). Compared with developed regions, governments in poor regions have greater control over scarce resources. To obtain the resources necessary for enterprise development, poor entrepreneurs rely more on government relations for resource acquisition to survive and develop (Cai et al., 2022). Additionally, the institutional environment of poor areas frequently has many gaps (Sinkovics et al., 2014), and the relationship with government departments allows poor entrepreneurs to obtain government support to avoid unnecessary administrative interventions (Faye and Niehaus, 2012). As one of the most important stakeholders of poor entrepreneurs, the government plays a significant role in their entrepreneurial activities.

The opportunity co-creation of poor entrepreneurs and the government refers to the use of knowledge, skills, and networks by poor entrepreneurs with government assistance providing advice, resource supply, and network to jointly develop and create entrepreneurial opportunities. The Chinese government has extensively changed the institutional rules and policy norms that inhibit entrepreneurship in poor areas (Zhou et al., 2018), providing more opportunities in poor communities and encouraging the poor to start a business. During this process, poor entrepreneurs and the government jointly assume the role of “incubators” for entrepreneurial opportunities. The Chinese government sent the first secretaries and local cadres to poor villages. Focusing on “wisdom and ambition,” they introduced policies to poor households through visits and conducted interviews to understand the requirements and issues of poor entrepreneurs (Cai et al., 2021). In turn, they established a targeted “one helping one” support mechanism for industrial poverty alleviation projects, provided advice and consultations for identifying and developing entrepreneurial opportunities, and actively encouraged and supported poor households towards independent entrepreneurship. Poor entrepreneurs can master entrepreneurial knowledge and skills by participating in government training and mentoring programs (Davie et al., 2021), allowing them to promptly recognize entrepreneurial opportunities, develop entrepreneurial activities, and improve business performance. At the same time, poor entrepreneurs can take advantage of the rural financial services set up by the government to strengthen their ability to withstand risks (Guo et al., 2019), explore new entrepreneurial opportunities, obtain a competitive edge, and enhance entrepreneurial performance (Khan et al., 2019). Additionally, the social network provided by the government can, to a certain extent, compensate for the essential resources needed by poor entrepreneurs to launch a business (Xu et al., 2022) to help create entrepreneurial opportunities and improve entrepreneurial performance (Wierenga, 2020).

To summarize, opportunity co-creation can assist poor entrepreneurs in identifying and developing entrepreneurial opportunities, help obtain key resources needed for entrepreneurship, and foster the growth of their entrepreneurial activities, thereby realizing the creation of economic and social value. Therefore, the following hypothesis is proposed:

*H1*: Opportunity co-creation has a direct positive effect on entrepreneurial performance.

### 2.4. Opportunity beliefs in the relationship between opportunity co-creation and entrepreneurial performance

Opportunity belief refers to the individual belief formed by entrepreneurs after overcoming the ignorance of opportunity information in the environment (Shepherd et al., 2007). This concept comes from the models of entrepreneurial action, reflected in the following dimensions: (1) the perception of the degree of alignment between a specific supply means and a target market, and (2) the perception of the general feasibility of the opportunity (Grégoire et al., 2010b). Autio et al. (2013) proposed that opportunity beliefs can be expressed in two aspects: first-person opportunity belief and third-person opportunity recognition. The first-person opportunity belief is a subjective belief formed by entrepreneurs who overcome the lack of opportunity information in a dynamic environment and believe the new venture idea to be personally attractive and worth pursuing (McMullen and Shepherd, 2006; Scheaf et al., 2020; Canavati et al., 2021). Third-person opportunity recognition refers to the relatively rational opportunity assessment results formed by entrepreneurs based on the potentially favorable or unfavorable outcomes the individual envisions from launching an imagined venture in a specific context (Autio et al., 2013; Canavati et al., 2021). When the entrepreneur’s first-person opportunity belief matches the third-person opportunity recognition, the entrepreneurial action will be triggered (Autio et al., 2013; Gish et al., 2019).

The opportunity co-creation between entrepreneurs and the government enables the poor to form opportunity beliefs. In the stage of targeted poverty alleviation, the five levels of party secretaries in China have jointly concentrated on poverty alleviation and established one-to-one pairing support relationships with the poor. To overcome the poor’s lack of information on poverty alleviation policies, government cadres discuss the national poverty alleviation policies with the poor (Zhang et al., 2021). Additionally, the government cadres strengthen ideological support, reducing doubts regarding the feasibility and desirability of opportunities and enhancing confidence among the poor (Yang and Liu, 2021). This helps entrepreneurs experience less psychological uncertainty, turning them from being unaware of entrepreneurial opportunities to being aware of them.

The support team repeatedly visits poor households and eats and lives with them to gain a thorough understanding of their needs before providing them with targeted industrial assistance, skills training, microfinance, and other policy measures (Li et al., 2016; Yang and Liu, 2021). These policy measures enable poor entrepreneurs to broaden their social horizons, networks, and contacts. Furthermore, they also allow poor entrepreneurs to gradually discover their social value through improved social network interactions, recognize their relative resource advantages in society, and better grasp current entrepreneurial policies, entrepreneurial skills, social networks, and other entrepreneurial resources (Guo et al., 2019). Therefore, poor entrepreneurs can more accurately grasp the changing trends of the market environment and detect flaws in the existing technology and the target market. They can then use this to clarify the degree of alignment between the two, evaluate the external environment (Grégoire et al., 2010b), and asses the feasibility of the opportunity. In the process of overcoming ignorance and becoming aware of accessible opportunity, the entrepreneur gives the opportunity meaning, which helps establish opportunity belief. Therefore, the following hypothesis is proposed:

*H2*: Opportunity co-creation has a direct positive effect on opportunity beliefs.

Opportunity beliefs are the entrepreneur’s vision for the future, that is, the objectives that the entrepreneur may achieve after taking entrepreneurial actions (Wood et al., 2014). This vision is based on the entrepreneur’s objective assessment of the degree of alignment between a particular supply means and a target market, as well as the overall feasibility and desirability of the opportunity (Grégoire et al., 2010b). Entrepreneurs with a strong sense of opportunity beliefs conduct a rigorous and objective analysis of entrepreneurial opportunities. Although this analysis is likely to be influenced by irrational factors such as cognitive bias, it can somewhat lessen the entrepreneurs’ perceptions of risks and the likelihood of failure (Krueger and Dickson, 1994; Goel and Karri, 2006), which increases the likelihood of entrepreneurial success or performance improvement (Baluku et al., 2016). Even with obstacles in the entrepreneurial process, entrepreneurs with a strong opportunity belief can overcome them confidently (Zou et al., 2016). In other words, the entrepreneur’s opportunity belief can greatly promote the development of entrepreneurial activities, promoting entrepreneurial performance (Furlotti et al., 2020). Based on the discussion, the following hypothesis is proposed:

*H3*: Opportunity beliefs have a direct positive effect on entrepreneurial performance.

In conclusion, the opportunity co-creation between poor entrepreneurs and the government makes it easier to start a business (Jang et al., 2020), helping entrepreneurs accurately grasp the shifting market environment and available entrepreneurial resources (Guo et al., 2019) and reducing entrepreneurs’ doubts about the feasibility and desirability of opportunities (Pidduck et al., 2021). Additionally, a stronger opportunity belief can reduce entrepreneurs’ perceptions of risks and the possibility of failure, promoting the development of entrepreneurial activities, and thereby positively predicting entrepreneurial performance (Baluku et al., 2016). Therefore, the hypothesis is proposed as follows:

*H4*: Opportunity beliefs play a mediating role between opportunity co-creation and entrepreneurial performance.

### 2.5. The role of entrepreneurial action between opportunity co-creation and entrepreneurial performance

Entrepreneurial action refers to discovering or creating, developing, and leveraging entrepreneurial opportunities and ultimately creating new enterprise models and new corporate organizations (Jaffe et al., 1993; Ireland, 2003; Duane Ireland and Webb, 2007). Previous studies have concluded the considerable influence of entrepreneurs’ gender (Murphy et al., 2021), previous experience (Chen and Pan, 2019), national culture (Bogatyreva et al., 2019), and other factors on entrepreneurial action. Additionally, most of the literature has confirmed the influence of entrepreneurial action on entrepreneurial outcomes (Bird and Schjoedt, 2017). However, as one of the important bridges between entrepreneurial opportunity co-creation and entrepreneurial performance, the specific mechanism of entrepreneurial action remains unclear.

Poor entrepreneurs frequently struggle with various issues when starting a business, including a lack of market information, essential resources, knowledge, and skills (Alvarez and Barney, 2014; Sutter et al., 2019). Therefore, it is difficult for them to adopt corresponding strategies to respond to the ups and downs of market normality. At the same time, decades of “blood transfusion poverty alleviation” have resulted in some poor communities lacking endogenous motivation to get rid of poverty and relatively weak development abilities. They also have a serious “ideology of waiting, relying, and needing” (Cai et al., 2022) and face various issues, such as a lack of confidence and enthusiasm for entrepreneurship and an incapacity to support subsequent production and development. It is, therefore, extremely difficult to promote the entrepreneurial behavior of the poor.

These issues can be somewhat alleviated by the opportunity co-creation between poor entrepreneurs and the government. Specifically, the opportunity co-creation between the government and poor entrepreneurs has a significant impact on entrepreneurial knowledge, management skills and abilities, the perception and evaluation of entrepreneurial opportunities, and the bricolage and integration of entrepreneurial resources (Jang et al., 2020). Opportunity co-creation also plays a significant role in promoting entrepreneurial actions. First, through communication with poor entrepreneurs, government cadres gain an understanding of the entrepreneurial intentions, problems, and needs of the poor. They work with the poor to discover and perceive potential business opportunities in the market, create entrepreneurial opportunities, and assist them in joining the entrepreneurial team in a timely and accurate manner, ensuring the steady advancement of entrepreneurial action (Li et al., 2019). Second, the opportunity co-creation between poor entrepreneurs and the government directly influences people’s entrepreneurial knowledge, skills, and management level (Yang and Liu, 2021). By participating in the government’s entrepreneurship guidance services, impoverished people can gradually cultivate entrepreneurship-related knowledge and skills (Yang and Liu, 2021), and increase their understanding of entrepreneurial opportunities, to lessen uncertainty (McMullen and Shepherd, 2006). This is conducive to stimulating and promoting the cultivation and development of entrepreneurial spirit represented by entrepreneurial behavior and encourages the poor to carry out entrepreneurial activities. Finally, the opportunity co-creation with the government can enable poor entrepreneurs to promptly obtain preferential entrepreneurial policy support such as microfinance (Zhou et al., 2018). As a result, the poor can acquire entrepreneurial resources and competitive advantages at a lower cost and faster speed, reducing entrepreneurial transaction costs and operating risks (Jang et al., 2020), thereby increasing their likelihood of entrepreneurship and ultimately effectively promoting the generation of entrepreneurial action. Therefore, the following hypothesis is proposed:

*H5*: Opportunity co-creation has a direct positive effect on entrepreneurial actions.

The existing literature has confirmed the positive effect of entrepreneurial action on entrepreneurial performance. On the one hand, continuous entrepreneurial actions can assist entrepreneurs in identifying the appropriate products, services, markets, or organizational forms (Mathias et al., 2015; Giones et al., 2020) and adapt as quickly as possible to the changing external environment, giving start-ups better adaptation mechanisms and competitive advantages to start-ups (Ferrier et al., 1999). On the other hand, the entrepreneurial behavior of entrepreneurs will promote the breakthrough innovation of enterprises; entrepreneurial behavior provides a channel for the overflow and transfer of knowledge, information, and technology, which contributes to the innovation and upgrading of enterprises and improves the innovation performance of entrepreneurship (Piñeiro-Chousa et al., 2020). Therefore, the following hypothesis is proposed:

*H6*: Entrepreneurial actions have a direct positive effect on entrepreneurial performance.

The opportunity co-creation between poor entrepreneurs and the government can enhance the perception and evaluation of entrepreneurial opportunities by poor entrepreneurs (Li et al., 2019), increase mastery of entrepreneurial knowledge, skill, and management capabilities (Yang and Liu, 2021), and support in the acquisition and integration of entrepreneurial resources (Wu et al., 2022a). This will enable entrepreneurs to promptly capture potential business opportunities in the market and obtain a competitive advantage with lower transaction costs and business risks (Jang et al., 2020), thereby promoting the development of entrepreneurial action. Additionally, good entrepreneurial action contributes to the innovation and upgrading of enterprises, increasing profitability, margins, and market share (Donbesuur et al., 2020) and enhancing entrepreneurial performance. Based on these arguments, the hypothesis is proposed as follows:

*H7*: Entrepreneurial actions play a mediating role between opportunity co-creation and entrepreneurial performance.

### 2.6. Opportunity beliefs and entrepreneurial actions

Opportunity beliefs and entrepreneurial actions are indispensable links in entrepreneurial activity. Shane and Venkataraman (2000) pointed out that opportunity belief is a prerequisite for entrepreneurial action. A strong opportunity belief demonstrates that entrepreneurs make a rigorous and objective assessment of the market environment and other factors, as well as a positive outlook on the outcomes of their ventures. Opportunity belief reflects the different evaluations of potential entrepreneurial opportunities. Its formation has two essential factors: (1) the opportunity exists and is perceived, and (2) the opportunity is feasible and desirable (McMullen and Shepherd, 2006).

When poor entrepreneurs and the government jointly create an entrepreneurial opportunity, they would have to assess its feasibility and desirability and thoroughly consider whether they can obtain, process, and integrate entrepreneurial resources and invest resources to create value (Haynie et al., 2009; Pidduck et al., 2021). With poverty alleviation policies and the assistance of poverty alleviation cadres, the poor can generate sufficient knowledge, have increased motivation to start a business, and form a third-person opportunity belief in jointly creating entrepreneurial opportunities with the government. Poor entrepreneurs become better equipped to recognize entrepreneurial opportunities in the environment (McMullen and Shepherd, 2006).

Once the third-person opportunity belief is formed, entrepreneurs have to overcome doubts to develop a first-person opportunity belief and produce a strong belief in “opportunity for me” (Pidduck et al., 2021). Doubt is created by entrepreneurial risk, uncertainty, and ambiguity. Individual doubt can significantly affect the formation of opportunity beliefs and stymie entrepreneurial action (McMullen and Shepherd, 2006; Shepherd et al., 2007). With government assistance, impoverished people can overcome ignorance, reduce doubt, and form a third-person opportunity belief and a first-person opportunity belief. The process enhances their confidence in getting rid of poverty and promotes their willingness to start a business, which spawns entrepreneurial actions (Wahid et al., 2021). As a result, a strong opportunity belief is more likely to motivate entrepreneurs to pursue entrepreneurial opportunities or other goals and promote the growth of entrepreneurial activities. Therefore, the following hypothesis is proposed:

*H8*: Opportunity beliefs have a direct positive impact on entrepreneurial actions.

In conclusion, a strong opportunity belief can assist entrepreneurs in overcoming doubts about the feasibility and desirability of opportunities (McMullen and Shepherd, 2006; Shepherd et al., 2007), thus providing psychological support for entrepreneurial action. Active entrepreneurial actions can assist entrepreneurs in adapting to the changing external environment as soon as possible, contribute to enterprise innovation and upgrading, and bring better adaptation mechanisms and competitive advantages to start-ups (Ferrier et al., 1999) to improve the innovation performance of entrepreneurship. Therefore, the hypothesis is proposed as follows:

*H9*: Opportunity beliefs and entrepreneurial actions together provide a mediating role between opportunity co-creation and entrepreneurial performance.

**Figure 1:**
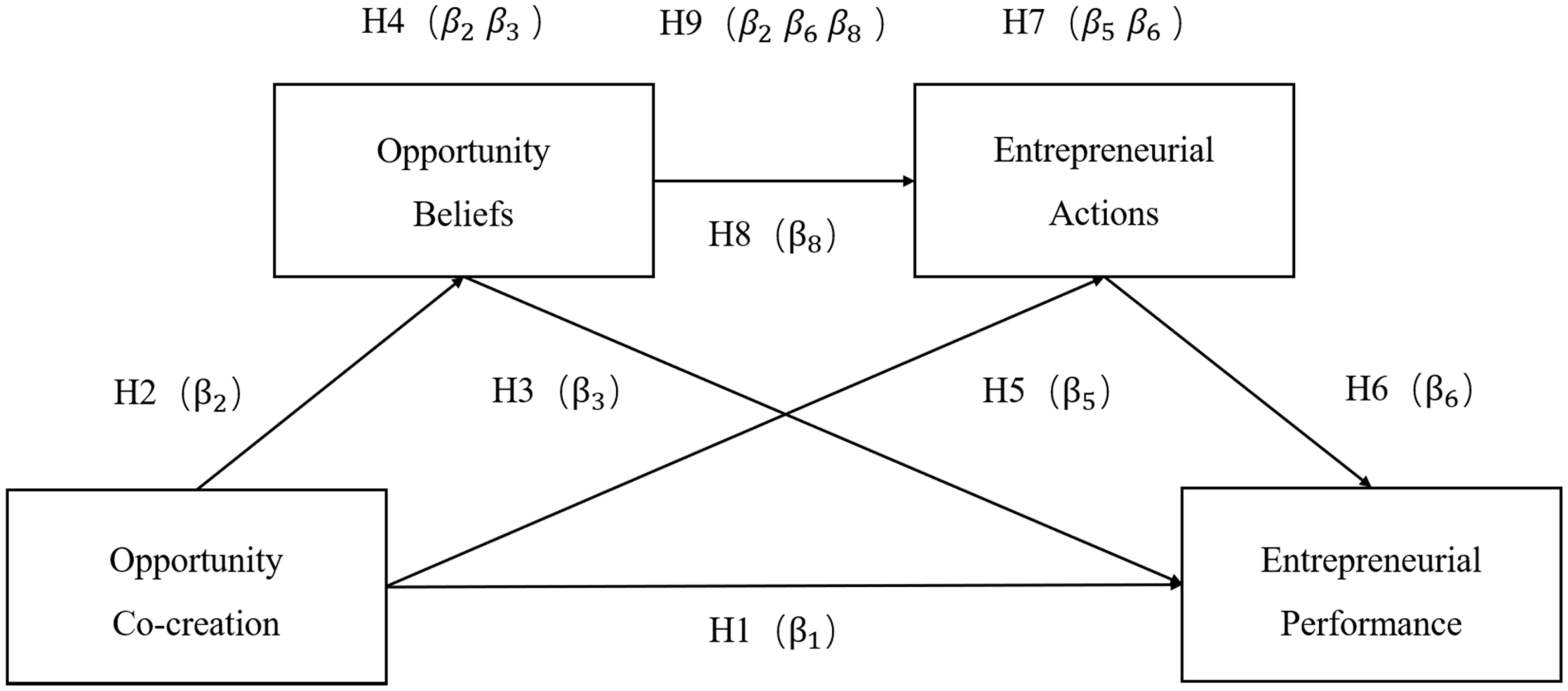
Research model. Italicized terms indicate mediating effects.

The hypothesis model in this study is a chain multiple mediation model using opportunity co-creation as the antecedent variable, opportunity beliefs and entrepreneurial actions as the mediators, and entrepreneurial performance as the outcome variable (see Figure 1). There are three intermediary paths: (1) H4:opportunity co-creation → opportunity beliefs → entrepreneurial performance (*β*_2_*β*_3_); (2) H7:opportunity co-creation → entrepreneurial actions → entrepreneurial performance (*β*_5_*β*_6_); and (3) H9:opportunity co-creation → opportunity beliefs → entrepreneurial actions → entrepreneurial performance (*β*_2_*β*_6_*β*_8_).

## 3. Methods

### 3.1. Scale design

The questionnaire design was based on pertinent scales developed and applied in previous studies. The variables were adjusted to fit the Chinese circumstance while maintaining semantic integrity. A 7-point Likert scale was used for measurement. Based on Sun and Im (2015) and Liu et al. (2019a), opportunity co-creation was measured using the following: (1) “The government or poverty alleviation cadres have established a relationship of trust with my business”; (2) “Poverty alleviation cadres have much face-to-face direct communication with me”; (3) “The government or poverty alleviation cadres encourage us to find ways to start a business”; (4) “The government or poverty alleviation cadres can exert influence on peers”; and (5) “The government or poverty alleviation cadres help my business solve problems arising from the product or service.”

Opportunity beliefs were measured using four items adapted from three established measures(Grégoire et al., 2010a,b; Grégoire and Shepherd, 2012). The items are: (1) “The company’s products/technologies can be used to solve foreseen market problems”; (2) “The company’s products/technologies can be used to solve foreseen market problems”; (3) “The functions of the company’s products/technologies are ‘matched’ with the foreseen market demand”; and (4) “The company’s products/technologies have been developed to make money in the foreseen market.”

Based on prior theoretical work by Dimov (2007b), entrepreneurial actions were assessed using the following four metrics: (1) “I discuss the opportunity with potential investors”; (2) “I discuss the opportunity with friends, family, or poverty alleviation officials”; (3) “I seek potential partners for exploiting this opportunity”; and (4) “I invest a certain amount of money in researching the viability of the opportunity.”

Entrepreneurial performance, measured using the scale by Li and Atuahene-Gima (2001), was evaluated through five items: (1) “The cost of each business transaction of the enterprise is lower than last year”; (2) “The company is quite satisfied with its performance internally”; (3) “The company has more cash flow than its competitors.”; (4) “The output value of new products accounts for a large proportion of total sales.”; and (5) “The company has applied for a large number of patents.”

At the individual level, we controlled entrepreneurs’ gender because entrepreneur’s gender has been shown as an important determinant of business performance (Lim and Suh, 2019). We also controlled for entrepreneur’s ethnicity because this variable has been proposed to explain variance in entrepreneurial performance (Howell, 2019). With regard to the organization level data, we controlled for enterprise size (with the number of employees as a measure), that seem to have an impact on the entrepreneurial performance (García-Sánchez et al., 2018). Further, we controlled the age of business because previous studies have suggested that the age of business can affect the business outcome (Coad et al., 2017). Before the questionnaire was formally finalized and researched, we discussed it with experts and scholars in the field of entrepreneurship poverty reduction to confirm its validity and reliability. Following numerous group discussions on experts’ opinions, an initial questionnaire draft was constructed. Then, 20 entrepreneurs from contiguous areas with dire poverty in China’s Wuling Mountains in China were selected for the pretest. The respondents, who were all senior managers with at least 3 years of work experience (such as general manager, chairman or boss), were asked to evaluate the questionnaire’s relevance, order, linguistic expression, and clarity. Based on their comments and suggestions, vague and misleading statements and redundant content were removed, and the formal questionnaire was created.

### 3.2. Data collection

The sample framework for this study involved entrepreneurs in the Wuling Mountains in China. Covering four provinces and cities (i.e., Hubei, Hunan, Chongqing, and Guizhou), the study area has a high concentration of ethnic minorities and poor people and has been an early pilot area for poverty alleviation in China. Initially, a purposive sampling within the social network of the research team dedicated to poverty issue in the area was conducted to recruit potential interviewees, before the snowball sampling to include a wider range of interview candidates. The questionnaires were handed out between April 2021 and June 2021. To collect more useful data as possible, we first contacted the entrepreneurs within the sampling framework, explained the purpose of the study, and asked for their cooperation and assistance. After obtaining permission, we went on site to conduct the survey. For those time-constrained, the questionnaires were distributed online for them to answer at their convenience. To ensure that the questionnaires were properly answered and fully completed, we double-checked each questionnaire and contacted the respondents with unclear markings or incomplete answers *via* phone or email. Out of the 500 questionnaires sent out, 331 were retrieved. Through a quality check of the questionnaires, 330 valid samples were finally obtained, with an effective recovery rate of 66%. Table 1 summarizes the descriptive statistics of the entrepreneurs surveyed in this study.

**Table 1:** Descriptive statistics of the samples.

Sample characteristics	Type	Frequency	Proportion(%)
Entrepreneur gender	Male	197	59.7
	Female	133	40.3
Entrepreneur ethnicity	Han Chinese	151	45.8
	Minorities	179	54.2
Enterprise size	<10	227	68.8
	10–49	56	17.0
	50–99	24	7.3
	100–499	13	3.9
	>500	10	3.0
Age of business	<3 years	161	48.8
	3–8 years	100	30.3
	>8 years	69	20.9

## 4. Data analysis and hypothesis testing

### 4.1. Common method bias analysis

Because the variables in this study were all self-rated by the respondents, common method deviation may emerge. To address this, we used statistical testing methods to assess if common method bias posed a risk to the analysis and interpretation of data. Using Harman single-factor test, the result revealed four factors with eigenvalues larger than 1, accounting for 70.03% of the variance explained, with the first principal component accounting for 37.32% of the variance (threshold of 40%). This suggests that common method bias was not a concern and would not significantly affect the relationships between variables.

### 4.2. Reliability and validity analysis

The reliability test and confirmatory factor analysis were performed using SPSS22.0 and Mplus8 software. First, we used SPSS22.0 for exploratory factor analysis (EFA). The KMO test statistics (Feng and Chen, 2020) and Bartlett’s Test of Sphericity (Liu et al., 2019b) were used as evaluation indexes to determine whether factor analysis could be carried out. Principal component analysis (PCA) was adopted to extract factors with eigenvalues greater than 1, and the maximum variance method was used for factor rotation. The results of the EFA showed that the KMO value for each scale was greater than 0.8, as shown in Table 2. The significance probability of the Bartlett test statistical value was 0.000, meaning that all variables were suitable for factor analysis. For all measurement scales, a factor was extracted; the factor loading for each item was between 0.645 and 0.923, which is within the acceptable range of 0.500–0.950. Additionally, the cumulative explanatory degree of the scale exceeded the 60% standard, indicating that the scale has good validity.

**Table 2:** Results of the confirmatory factor analysis.

Items	Factor loading	Items	Factor loading
Opportunity co-creation		Entrepreneurial Action	
OCC1	0.809	EA1	0.762
OCC2	0.923	EA2	0.668
OCC3	0.804	EA3	0.865
OCC4	0.668	EA4	0.776
OCC5	0.886	Entrepreneurial Performance	
Opportunity beliefs		EP1	0.718
OB1	0.824	EP2	0.645
OB2	0.787	EP3	0.785
OB3	0.821	EP4	0.842
OB4	0.652	EP5	0.66

We then used Cronbach’s alpha for the reliability test. The Cronbach’s alpha for all variables was greater than 0.8 (see Table 3), indicating high reliability of items. The average variance extracted (AVE) for all variables was higher than 0.5, ranging between 0.539 and 0.677, while the composite reliability (CR) values were above 0.8, ranging from 0.852 to 0.912; the results suggest strong convergence validity. Finally, correlation analysis was conducted; the correlation coefficients among all variables were statistically significant at different statistical levels. All the square root AVE values exceeded the correlation coefficient across factors, indicating excellent discriminant validity.

**Table 3:** Results of the reliability and validity analysis.

Variable	α	Mean	SD	CR	AVE	OC	OB	EA	EP
OC	0.854	4.409	1.372	0.912	0.677	0.823			
OB	0.707	4.976	0.981	0.852	0.539	0.255**	0.734		
EA	0.909	5.113	1.101	0.856	0.599	0.436**	0.406**	0.774	
EP	0.851	4.472	1.012	0.853	0.594	0.379**	0.383**	0.473**	0.771

** *p* < 0.01. Square root of AVEs in boldface on the diagonal of the matrix.

### 4.3. Hypothesis testing

**Figure 2:**
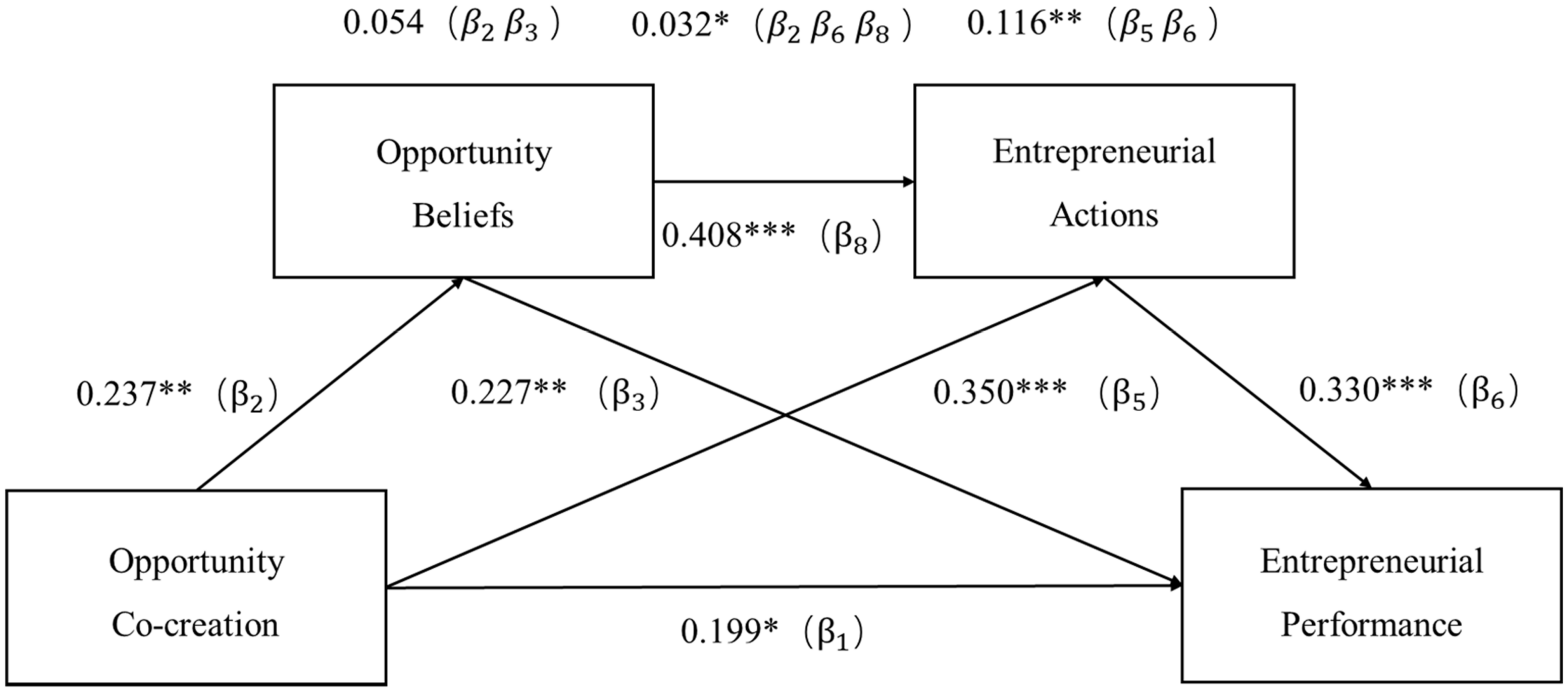
The output of the hypothesis model. Italics indicate mediating effects. *p < 0.05, **p < 0.01, and ***p < 0.001.

We conducted the intermediary analysis based on the structural equation in Mplus8 to test the intermediary effect of opportunity beliefs and entrepreneurial actions, combining the non-parametric percentile Bootstrap method (bootstrap = 5,000) with a 95% bias confidence interval (CI). In the Bootstrap program, a total of 5,000 repeated samples was set, and a 95% confidence interval was evaluated. If the confidence interval includes 0, the effect is not significant; if the confidence interval does not include 0, the effect is statistically significant. The model fit indexes suggest that the hypothesized structural model fitted well on the whole, with χ^2^ = 405.492, df = 197, χ^2^/df = 2.058, RMSEA = 0.057, SRMR = 0.063, CFI = 0.938, and TLI = 0.929. The output of the hypothesis model is shown in Figure 2, and the hypothesis test results are shown in Table 4.

**Table 4:** Results of the hypothesis testing.

Influence path	Direct effect	95% confidence interval	Indirect effect	95% confidence interval
Gender → EP	−0.108*	[−0.204, −0.012]	-	-
Ethnicity → EP	−0.001	[−0.098, 0.098]	-	-
Enterprise Size → EP	0.025	[−0.081, 0.132]	-	-
Age of Business → EP	−0.110*	[−0.212, −0.014]	-	-
OC → EP	0.199*	[0.048, 0.354]	0.201***	[0.117, 0.304]
OC → OB	0.237**	[0.093, 0.380]	-	-
OB → EP	0.227**	[0.063, 0.397]	0.135**	[0.062, 0.239]
OC → EA	0.350***	[0.213, 0.483]	0.097**	[0.037, 0.178]
EA → EP	0.330***	[0.111, 0.495]	-	-
OB → EA	0.408***	[0.247, 0.572]	-	-

* *p* < 0.05;

** *p* < 0.01;

*** *p* < 0.001.

#### 4.3.1. Direct effect test

(1) Hypothesis H1 proposes that opportunity co-creation between poor entrepreneurs and the government is positively associated with good entrepreneurial performance. As shown in Table 4, in the influence path from opportunity co-creation to entrepreneurial performance, the 95% confidence interval for the direct effect is [0.048, 0.354], excluding 0, suggesting that opportunity co-creation has a significant direct positive effect on entrepreneurial performance (*β*_1_ = 0.199, *p* = 0.011). Thus, hypothesis H1 is verified. Among the control variables, entrepreneurs’ gender significantly negatively affected entrepreneurial performance (*β* = −0.108, *p* = 0.029), indicating that male entrepreneurs exhibited better entrepreneurial performance than female entrepreneurs. The age of business significantly negatively influenced entrepreneurial performance. The values (*β* = −0.110, *p* = 0.029) highlight that the age of business had a negative impact on the entrepreneurial performance. Finally, entrepreneurs’ ethnicity and enterprise size had no significant influence on entrepreneurial performance.
(2) Hypothesis H2 proposes that opportunity co-creation has a positive impact on opportunity beliefs. As shown in Table 4, in the influence path from opportunity co-creation to opportunity beliefs, the 95% confidence interval for the direct effect is [0.093, 0.380], excluding 0. This suggests that opportunity co-creation has a significant direct positive effect on opportunity beliefs (*β*_2_ = 0.237, *p* = 0.001), thus confirming hypothesis H2.
(3) Hypothesis H3 purports that opportunity beliefs positively contribute to entrepreneurial performance. Based on the influence path from opportunity beliefs to entrepreneurial performance in Table 4, the 95% confidence interval for the direct effect is [0.063, 0.397, which excludes 0. The results indicate that opportunity beliefs have a significant direct positive effect on entrepreneurial performance (*β*_3_ = 0.227, *p* = 0.008). Thus, hypothesis H3 is verified.
(4) Hypothesis H5 suggests that opportunity co-creation between poor entrepreneurs and the government is positively associated with entrepreneurial actions. According to Table 4, in the influence path from opportunity co-creation to entrepreneurial actions, the 95% confidence interval for the direct effect is [0.213, 0.483], excluding 0, indicating that opportunity co-creation has a significant direct positive effect on entrepreneurial actions (*β*_5_ = 0.350, *p* < 0.001). Therefore, hypothesis H5 is confirmed.
(5) Hypothesis H6 argues that entrepreneurial actions positively affect entrepreneurial performance. As shown by the influence path from entrepreneurial actions to entrepreneurial performance in Table 4, the 95% confidence interval for the direct effect is [0.111, 0.495], excluding 0, indicating that entrepreneurial actions have a significant direct positive effect on entrepreneurial performance (β_6_ = 0.330, *p* < 0.001); thus, hypothesis H6 is validated.
(6) Hypothesis H8 purports that opportunity beliefs positively contribute to entrepreneurial actions. According to Table 4, in the influence path from opportunity beliefs to entrepreneurial actions, the 95% confidence interval for the direct effect is [0.247,0.572], excluding 0. This means that opportunity beliefs have a significant direct positive effect on entrepreneurial actions (*β*_8_ = 0.408, *p* < 0.001), verifying hypothesis H8.

#### 4.3.2. Indirect effect test

The bootstrapping results in Table 4 indicate that in the influence path from opportunity co-creation to entrepreneurial performance, the 95% confidence interval for indirect effect is [0.117, 0.304], excluding 0. This means that the indirect effect between opportunity co-creation and entrepreneurial performance is statistically significant. In the influence path from opportunity beliefs and entrepreneurial performance, the 95% confidence interval for indirect effect is [0.062, 0.239], which does not contain 0, suggesting that the indirect effect between opportunity beliefs and entrepreneurial performance is significant. In the influence path from opportunity co-creation to entrepreneurial actions, the 95% confidence interval for indirect effect is [0.037, 0.178], excluding 0. This suggests that the indirect effect between opportunity co-creation to entrepreneurial actions is significant.

In summary, opportunity beliefs and entrepreneurial action jointly play a chain-mediating role between opportunity co-creation and entrepreneurial performance. Their total mediating effect is 0.021 (*β_2_β_3_* + *β_5_β_6_* + *β_2_β_6_β_8_*), accounting for 50.25% of the total effect. The mediating effect of opportunity beliefs alone was 0.054 (*β_2_β_3_*), accounting for 13.5% of the total effect; thus, Hypothesis H4 is validated. The mediating effect of entrepreneurial action alone was 0.116 (*β_5_β_6_*), accounting for 29% of the total effect. Hypothesis H7 has been verified. The chain-mediating effect of opportunity beliefs and entrepreneurial action was 0.032 (*β_2_β_6_β_8_*), accounting for 8% of the total effect. Hypothesis H9 is confirmed.

## 5. Discussion and conclusion

### 5.1. Discussion

We constructed a hypothesis model and conducted empirical analysis on 330 poor entrepreneurs in contiguous areas of dire poverty in the Wuling Mountains, China. The results show that opportunity co-creation has a positive effect on entrepreneurial performance *via* a variety of mechanisms, including (1) opportunity co-creation → entrepreneurial performance, (2) opportunity co-creation → opportunity beliefs → entrepreneurial performance, (3) opportunity co-creation → entrepreneurial action → entrepreneurial performance, (4) opportunity co-creation → opportunity beliefs → entrepreneurial action → entrepreneurial performance. The findings suggest that opportunity co-creation has a direct and positive effect on entrepreneurial performance, as well as an indirect positive impact *via* the partly mediating effect of opportunity beliefs and entrepreneurial actions. Opportunity beliefs and entrepreneurial actions work in tandem to mediate the relationship between opportunity co-creation and entrepreneurial performance; the chain-mediating effect was 0.201, which accounts for 50.25% of the total effect and explains the majority of the variance between opportunity co-creation and entrepreneurial performance.

First, opportunity co-creation has a significant positive impact on entrepreneurial performance. Opportunity co-creation with the government can allow entrepreneurs in poor areas to overcome the lack of entrepreneurial opportunities. Previous studies have concluded that the opportunity co-creation process between MFIs and the government can help reduce interest rates for the poor (Alvarez and Barney, 2014; Sun and Im, 2015). This study further examined the significant positive impact of opportunity co-creation on the entrepreneurial performance of the poor and employed opportunities co-creation as a key element in analyzing entrepreneurship and poverty reduction. The opportunity co-creation between poor entrepreneurs and government refers to the use of knowledge, skills and networks acquired with government assistance to provide advice, resource supply, and network support for identifying and developing entrepreneurial opportunities (Guo et al., 2019; Davie et al., 2021). This would help provide some of the essential resources poor entrepreneurs need to start a business (Xu et al., 2022) to develop and create entrepreneurial opportunities jointly with the government, assisting enterprises to promptly adapt to the market environment and improve entrepreneurial performance.

Second, opportunity beliefs significantly mediate the relationship between opportunity co-creation and entrepreneurial performance. Having a strong opportunity belief is crucial for poor entrepreneurs to build their core competitiveness. As the degree of opportunity co-creation between poor entrepreneurs and the government deepens, entrepreneurs become cognizant of poverty alleviation policies, improve their existing entrepreneurial skills, social networks, and other entrepreneurial resources, understand changing trends in the market (Guo et al., 2019), and perceive defects in the existing technology and target market (Grégoire et al., 2010b). After entrepreneurs are able to overcome their ignorance and doubt of the environment and assess entrepreneurial opportunities, their views on risks and the possibility of failure are reduced to some extent (Krueger and Dickson, 1994; Goel and Karri, 2006), which helps increase the likelihood of entrepreneurial success (Baluku et al., 2016).

Third, entrepreneurial action plays a significant role in mediating the relationship between opportunity co-creation and entrepreneurial performance; this means that entrepreneurial action is vital for turning opportunities into performance. The findings demonstrate that the opportunity co-creation with the government can help poor entrepreneurs obtain the essential knowledge, skills, and resources, decrease the costs and risks of starting a business (Jang et al., 2020; Yang and Liu, 2021), and encourage entrepreneurial action. In addition, opportunity co-creation can help enterprises quickly adapt to the rapidly changing external environment, create competitive advantages (Ferrier et al., 1999), and enhance entrepreneurial performance.

Fourth, opportunity beliefs and entrepreneurial actions play multiple mediating roles between opportunity co-creation and entrepreneurial performance. Opportunity beliefs and entrepreneurial action are indispensable links in entrepreneurial activities. We found that opportunity belief is the driving force behind entrepreneurial action, and the two working together can boost entrepreneurial performance. The results demonstrate that more focus should be given to assessing and exploring entrepreneurial opportunities and developing entrepreneurial action after the opportunity evaluation.

### 5.2. Theoretical contribution

The main theoretical contributions of this study are as follows:

First, this study provides a useful addition to the literature on poverty reduction. Using poor entrepreneurs in contiguous areas of dire poverty in the Wuling Mountains as respondents is a useful addition to the sample of studies on poverty reduction. Over the past few decades, China has significantly reduced poverty, adopting poverty reduction strategies with distinct Chinese characteristics. This study examining opportunity co-creation between poor Chinese entrepreneurs and Chinese government agencies by empirical study of 330 entrepreneurs in contiguous areas of dire poverty in the Wuling Mountains in China provides a better understanding of the orientation and path of entrepreneurial strategies.

Second, clarifying the impact of opportunity co-creation on entrepreneurial performance provides scientific guidance for poverty reduction. Past studies on entrepreneurial opportunities have focused on the “discovery view” or the “creation view,” ignoring interactions between various elements of entrepreneurial opportunities and limiting the overall understanding of entrepreneurial opportunity sources. Previous studies pointed out that entrepreneurial opportunities result from entrepreneurs, markets, and environments creating new means-ends relationships through various interactive activities (Busenitz et al., 2003; Johnson and Schaltegger, 2020). Entrepreneurial opportunities evolve from the co-creation with stakeholders (Alvarez and Barney, 2014) who can contribute to poverty alleviation driven by their social mission (Sun and Im, 2015). A new perspective of opportunity co-creation is introduced. The positive effect of opportunity co-creation on entrepreneurial performance is verified, providing new ideas for entrepreneurial poverty reduction research. This study addresses some research gaps in entrepreneurial opportunity co-creation, exploring the processes and mechanisms of opportunity co-creation between poor entrepreneurs and the government. Compared with non-poor entrepreneurs, poor entrepreneurs are mostly situated in remote communities (e.g., rural areas), which can be challenging to stay updated on market changes (Ring et al., 2010). At the same time, the poor often lack a comprehensive knowledge base (Anderson and Obeng, 2017), making it difficult to discover, identify and construct entrepreneurial opportunities. Considering the constraints of poor entrepreneurs, this study explored how entrepreneurs could overcome various problems in the entrepreneurial process through opportunity co-creation with the government, providing new insights for studying entrepreneurial opportunities and scientific theoretical guidance for entrepreneurship for poverty alleviation.

Third, investigating the inherent logic of opportunity beliefs and entrepreneurial actions helps untangle the “black box” relationship between opportunity co-creation and entrepreneurial performance. Existing research on the interaction between entrepreneurial opportunities, subjects, and the environment remains insufficient (Ramoglou and Gartner, 2022), leading to a poor understanding of the various interaction mechanisms, antecedents and effects. While some studies have explored the role of opportunity beliefs and entrepreneurial actions in entrepreneurial activities (Pidduck et al., 2021), few have thoroughly examined their links and differences. Based on the framework of“Beliefs-Actions-Results” (BAR; Foss and Klein, 2020), this study innovatively introduces opportunity beliefs and entrepreneurial actions as key research elements into the research model of opportunity co-creation. The results indicate that opportunity belief has a significant positive contribution to entrepreneurial actions and functions as a chain-mediating link in the relationship between opportunity co-creation and entrepreneurial performance. The findings establish the relationship between opportunity belief and entrepreneurial action and reveal opportunity co-creation pathways affecting entrepreneurial performance.

### 5.3. Practical implications

The research findings have various practical implications for entrepreneurship and poverty reduction. First, elucidating the potential pathway of poverty reduction through entrepreneurship, this study may aid developing countries in expanding their entrepreneurship initiatives, particularly in underprivileged areas, and in enhancing policies that would encourage long-term sustainable economic growth. Second, Poor communities should take full advantage of entrepreneurial opportunities provided by government assistance, and poor entrepreneurs should frequently interact with institutions and the government to jointly create entrepreneurial opportunities (Deng et al., 2022), as evidence from this study suggests that poor entrepreneurs could effectively promote the transformation of entrepreneurial ideas into realistic, objective opportunities during their interactions with government agencies. Third, in their interactions with the government, poor entrepreneurs should focus on validating and enhancing their business ideas to strengthen their own opportunity beliefs, while avoiding any possible risks brought on by information asymmetry.

### 5.4. Limitations and future work

The following limitations should be addressed in future research. First, this study investigated the poor entrepreneurs in contiguous impoverished areas in the Wuling Mountains in China. The results should be compared with those in other regions of China or similar areas. Subsequent studies should verify the findings by focusing on the experience of entrepreneurial poverty alleviation in other countries to increase the validity of the study’s conclusions. Second, the findings indicate that opportunity beliefs and entrepreneurial actions alone are insufficient to fully account for the impact of opportunity co-creation on entrepreneurial performance. Subsequent studies should explore other mediating variables between opportunity co-creation and entrepreneurial performance. Third, this study did not discuss the extent of opportunity co-creation’s impact on entrepreneurial performance. Future studies should examine the impact of entrepreneurs’ personality traits on the relationship between opportunity co-creation and entrepreneurial performance to better understand the impact of opportunity co-creation on entrepreneurial performance.

### 5.5. Conclusion

This study set out to examine where the poor’s entrepreneurial opportunities emerge from and how opportunity co-creation relates to the success of poor entrepreneurs. Based on a review of the literature on entrepreneurial opportunity, opportunity co-creation, opportunity beliefs, entrepreneurial action, and their relationship with entrepreneurial performance, we proposed that opportunity co-creation between poor entrepreneurs and the government would positively impact poor entrepreneurs’ opportunity beliefs and entrepreneurial actions, thereby affecting entrepreneurial performance. A survey was then carried out with entrepreneurs used to be poverty-stricken population in the Wuling Mountain Region. Structural equation analysis reveals direct positive impacts of opportunity co-creation between poor entrepreneurs and the government on entrepreneurial performance, opportunity co-creation on opportunity beliefs and entrepreneurial actions, opportunity beliefs on entrepreneurial actions, as well as opportunity beliefs and entrepreneurial actions on entrepreneurial performance. On the other hand, the indirect effects of opportunity co-creation on entrepreneurial actions, and opportunity co-creation and opportunity beliefs on entrepreneurial performance are also significant. Taken together, these findings suggest that the effect of opportunity co-creation on entrepreneurial performance is chain-mediated by opportunity beliefs and entrepreneurial actions. The empirical findings in this study provide a deeper insight into the link between opportunity beliefs and entrepreneurial behavior, while offering opportunity co-creation solutions for poverty reduction through entrepreneurship.

## Data availability statement

The raw data supporting the conclusions of this article will be made available by the authors, without undue reservation.

## Ethics statement

Ethical review and approval were not required for the study on human participants in accordance with the local legislation and institutional requirements. The patients/participants provided their written informed consent to participate in this study.

## Author contributions

XC and YT: conceptualization. XC: methodology, software, writing—original draft preparation, and visualization. XC, YZ, and YT: validation and writing—review and editing. YZ and YT: investigation. YZ, HC, and YT: resources and supervision and data curation and funding acquisition. YT: project administration. All authors contributed to the article and approved the submitted version.

## Funding

This work was supported by National Natural science Foundation of China (Grant Nos. 72262008 and 72062010).

## Conflict of interest

The handling editor YZ declared a shared affiliation with the author YT at the time of review.

The remaining authors declare that the research was conducted in the absence of any commercial or financial relationships that could be construed as a potential conflict of interest.

## Publisher’s note

All claims expressed in this article are solely those of the authors and do not necessarily represent those of their affiliated organizations, or those of the publisher, the editors and the reviewers. Any product that may be evaluated in this article, or claim that may be made by its manufacturer, is not guaranteed or endorsed by the publisher.
